# Potential Antioxidant and Anti-Inflammatory Effects of *Spilanthes acmella* and Its Health Beneficial Effects: A Review

**DOI:** 10.3390/ijerph18073532

**Published:** 2021-03-29

**Authors:** Rohanizah Abdul Rahim, Putri Ayu Jayusman, Norliza Muhammad, Norazlina Mohamed, Vuanghao Lim, Nor Hazwani Ahmad, Sharlina Mohamad, Zuratul Ain Abdul Hamid, Fairus Ahmad, Norfilza Mokhtar, Ahmad Nazrun Shuid, Isa Naina Mohamed

**Affiliations:** 1 Pharmacology Department, Faculty of Medicine, Universiti Kebangsaan Malaysia, Cheras, Kuala Lumpur 56000, Malaysia; rohanizah@usm.my (R.A.R.); putri.ayujay@gmail.com (P.A.J.); norliza_ssp@ppukm.ukm.edu.my (N.M.); azlina@ppukm.ukm.edu.my (N.M.); 2 Advanced Medical and Dental Institute, Universiti Sains Malaysia, Bertam, Kepala Batas 13200, Malaysia; vlim@usm.my (V.L.); norhazwani@usm.my (N.H.A.); sharlina@usm.my (S.M.); 3 School of Materials and Mineral Resources Engineering, Engineering Campus, Universiti Sains Malaysia, NibongTebal 14300, Malaysia; srzuratulain@usm.my; 4 Anatomy Department, Faculty of Medicine, Universiti Kebangsaan Malaysia, Cheras, Kuala Lumpur 56000, Malaysia; apai.kie@gmail.com; 5 Physiology Department, Faculty of Medicine, Universiti Kebangsaan Malaysia, Cheras, Kuala Lumpur 56000, Malaysia; norfilza@ppukm.ukm.edu.my; 6 Faculty of Medicine UiTM, Sg Buloh 47000, Malaysia; anazrun@yahoo.com

**Keywords:** *Spilanthes acmella*, antioxidant, anti-inflammatory, iNOS, NF-κB, COX-2, MAPK

## Abstract

Oxidative stress and inflammation are two common risk factors of various life-threatening disease pathogenesis. In recent years, medicinal plants that possess antioxidant and anti-inflammatory activities were extensively studied for their potential role in treating and preventing diseases. *Spilanthes acmella* (*S. acmella*), which has been traditionally used to treat toothache in Malaysia, contains various active metabolites responsible for its anti-inflammatory, antiseptic, and anesthetic bioactivities. These bioactivities were attributed to bioactive compounds, such as phenolic, flavonoids, and alkamides. The review focused on the summarization of in vitro and in vivo experimental reports on the antioxidant and anti-inflammatory actions of *S. acmella*, as well as how they contributed to potential health benefits in lowering the risk of diseases that were related to oxidative stress. The molecular mechanism of *S. acmella* in reducing oxidative stress and inflammatory targets, such as inducible nitric oxide synthase (iNOS), transcription factors of the nuclear factor-κB family (NF-κB), cyclooxygenase-2 (COX-2), and mitogen-activated protein kinase (MAPK) signaling pathways were discussed. Besides, the antioxidant potential of *S. acmella* was measured by total phenolic content (TPC), total flavonid content (TFC), 2,2-diphenyl-1-picrylhydrazyl (DPPH), and superoxide anion radical scavenging (SOD) and thiobarbituric acid reactive substance (TBARS) assays. This review revealed that *S. acmella* might have a potential role as a reservoir of bioactive agents contributing to the observed antioxidant, anti-inflammatory, and health beneficial effects.

## 1. Introduction

Inflammation is a protective mechanism that aims to restore the integrity of damaged or threatened tissues from injury or infectious pathogens [1]. Meanwhile, chronic inflammation is a prolonged pathological condition characterized by mononuclear immune cell infiltration, tissue destruction, and fibrosis. It adversely affects cell function through excessive production of free radicals and depletion of antioxidants [2]. The overproduction of reactive oxygen species (ROS) from activated neutrophils and macrophages leads to tissue injury by damaging the macromolecules and lipid peroxidation of the membrane [3,4]. Various life-threatening diseases including cancer, neurodegenerative and cardiovascular diseases are associated with inflammation and oxidative stress.

Non-steroidal anti-inflammatory drugs (NSAIDs) are commonly prescribed as analgesics and to reduce inflammation in diseases such as rheumatoid arthritis, osteoporosis, and Alzheimer’s disease. However, NSAIDs are associated with side effects such as gastrointestinal bleeding and suppressed function of the immune system [5]. Therefore, attention has shifted to the search for natural anti-inflammatory agents that possess very few side effects [6].

Medicinal plants are important sources of therapeutically active compounds that serve as leads for developing novel drugs. About 52% of molecules from natural products or derived from natural products were approved between 1981 and 2014 by the US Food and Drug Administration (FDA) [7]. Newman and Crag [8], reported that 83% of the new chemical entities registered were from natural or products supported by natural products. Examples of the drugs were: (a) paclitaxel which is widely utilized in breast cancer treatment and isolated from the bark of *Taxus brevifolia* Nutt [7]; (b) quinine, which is an anti-malarial drug approved by the United States FDA in 2004, is isolated from the bark of *Cinchona succirubra* Pav. ex Klotzsch, and used for the treatment of malaria, fever, indigestion, mouth and throat diseases, and cancer; (c) pilocarpine, which is used for the treatment of chronic glaucoma and acute glaucoma is isolated from *Pilocarpus jaborandi* Holmes [9].

Since ancient times, disorders related to inflammatory conditions have been treated with plants or plant-derived formulations. Dias et al. [9] reported that the oil from *Cupressus sempervirens* L. and resin from *Commiphora* species were used to treat coughs, colds, and inflammation. *Parmelia omphalodes* (L.) Ach. is used traditionally to prevent foot inflammation. The most well-known anti-inflammatory agent of acetylsalicyclic acid (aspirin) and salicin was derived from a natural product, which was isolated from the bark of the willow tree, *Salix alba* L. [9]. Besides, many medicinal plants rich in antioxidants display protective effects against inflammation [10].

Plant extracts, formulations, and bioactive components of *Spilanthes* spp. have been documented to have a wide range of potential applications in pharmaceutical industries [11]. They are even sold over the counter and on the internet under various names besides being widely used in various cultures. Jansen [12], reported that the genus *Spilanthes* (*S.*) was first described in 1760 by Jacquin and contained gametic chromosome number of 16. According to the Hennigan cladogram, there are two main lines of genus *Spilanthes* evolution. The first line is *Spilanthes costata* Benth and the second line is *Spilanthes Leiocarpa* DC., *Spilanthes nervosa* Chodat, *Spilanthes paraguayensis* R. K. Jansen, and *Spilanthes urens*. The genus *Spilanthes* has 60 species, which is distributed throughout the tropical and subtropical regions of the world [13]. This herb is native to Brazil and widely distributed over the world, including America, North Australia, Borneo, Malaya, Africa, India, and Sri Lanka [11]. From biological perspectives and evaluation of traditional use, *Spilanthes acmella* (*S.acmella*) is one of the most studied genus species.

**Figure 1:**
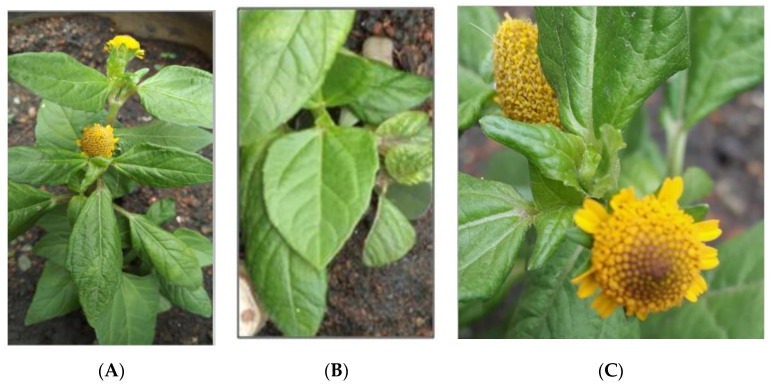
**(A**) *S. acmella* plant (**B**) *S.acmella* leaves (**C**) *S. acmella* flowers.

*S. acmella* (Figure 1) belongs to the Asteraceae family (Table 1), which is derived from the type of genus Aster, while ‘Compositae’ is an older name that is still valid for referring to the characteristic inflorescence [11]. The flower heads are either solitary or occur in compact or spreading inflorescences [11] (Figure 1C). Asteraceae is the largest family and the most diverse flowering plant family which comprises 24,000 to 30,000 species and 1600 to 1700 genera [14]. Its large-scale production as angiosperm phylogeny is present in Asterideae [15]. The three-marker phylogeny of Asteraceae includes rbcL, ndhF, and matK [14].

This herb is native to Brazil and popularly known as a toothache plant owing to its traditional use to relieve dental pain. It is cultivated throughout the year as an ornamental or medicinal plant. It can also be found in a damp area, at swamp margin, on rocks near the sea, or as a road-side weed [16]. It is an annual or short-lived prostrate plant with ascending cylindrical hairy stems and has no flower petals. All parts of the plant are unpleasantly bitter, with the flower heads being the most pungent part, causing a tingling sensation and numbness [17], itchiness, and salivation [18]. It has been used as a traditional medicine to treat various illnesses, mostly attributed to its secondary bioactive metabolites.

Abd Jalil et al. [19], reported that nowadays researchers and doctors are interested in traditional medicines due to their potential in treating diseases. Many diseases are associated with oxidative stress within the body system, such as osteoporosis [19], cancer [20], cardiovascular disease [21], and diabetes [22]. This oxidative stress is related to the antioxidant and anti-inflammatory activities of the body system. Anti-inflammatory drugs, such as nonsteroidal anti-inflammatory drugs (NSAIDs) have adverse effects on the gastrointestinal lining, coagulation of blood and renal systems, due partly to inhibition of housekeeping enzyme COX-1. Therefore, research on natural plants as alternative medicines should be explored comprehensively as alternative anti-inflammatory agents with minimal and no side effects.

This review is aimed at assessing the antioxidant and anti-inflammatory activities of *S. acmella* in reducing the risks of their related diseases. The outcome of this review revealed that *S. acmella* has potential as a bioactive agent with antioxidant and anti-inflammatory activities for health beneficial effects.

## 2. *Spilanthes acmella*

### 2.1. Traditional Medicinal Uses

Traditional medicine has remained the most affordable and easily accessible source of treatment since prehistoric times. Medicinal plants are still the most important health care source for the vast majority of the population around the world [23,24]. *S. acmella,* which can also be found worldwide, is widely used, traditionally in the tropics and subtropics countries, mainly India and South America. They are typically chewed to reduce toothache and relieve throat and gum infections, as well as to paralyze the tongue [25]. According to the dictionary of Malay Peninsula plant products, a decoction of this plant is taken internally as a diuretic and to resolve stones in the bladder, while a decoction of the roots is used as purgative. It is also used to prevent scurvy and stimulate digestion [26].

*S. acmella* is among the most common Amazonian medicinal plants used by the lay population of the Amazon basin for treating tuberculosis [27]. In India, the juice of *S. acmella* inflorescence is used to treat mouth ulcers [11]. In Western India, the flower head of *S. acmella* is a popular remedy for stammering in children. It is also used to treat dysentery and rheumatism [28]. Both leaves and flowers of *S. acmella* are traditionally used to treat leucorrhea by tribes in Bangladesh [29]. In Cameroon, *S. acmella* is used as a snakebite remedy and articular rheumatism treatment [30]. Apart from its medicinal uses, the Japanese use the flowers of this species as a spice for appetizers while its extract is used as a flavoring material for dentifrices and gum [31]. Meanwhile, the plant leaves are used as a spice in Brazilian cuisine to produce the characteristic tingling paraesthesia of the traditional regional dishes [25].

### 2.2. Phytochemical

**Figure 2:**
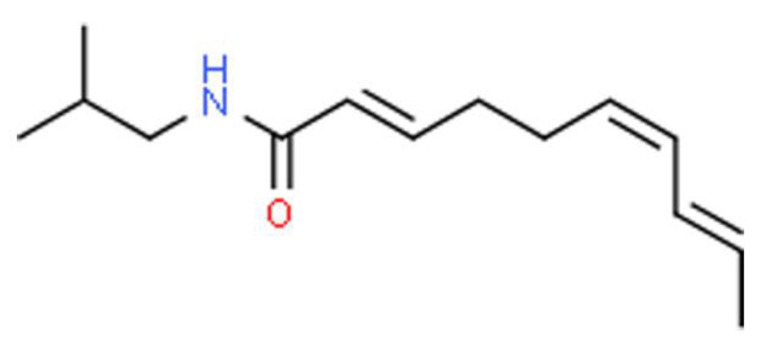
The chemical structure of *Spilanthol.*

Medicinal plants used in traditional medicine may contain various bioactive compounds, which could exert significant physiological actions on the human body. Phytochemically, *S. acmella* has been reported to contain isobutyl amide derivatives [32], amino acids, α- and β-amyrin esters, myricyl alcohol including sitosterol glucosides [33], and triterpenoid [34]. Asteracea is one of the eight plant families that has been documented to have alkamides as secondary metabolites [35]. Alkamides are considered as the most predominant phytochemicals present in genus *Spilanthes* and the most significant alkamides found is spilanthol (Figure 2).

Spilanthol, which is *N*-isobutylamide, is the major pungent compound in *S. acmella* responsible for various biological activities. The structure of spilanthol is elucidated as (2*E*, 6*Z*, 8*E*)-N-isobutylamide-2,6,8-decatrienamide [36]. Gas chromatography-mass spectrometry (GC-MS) analysis revealed that spilanthol is present in the mother plant, flower heads, and in vitro plantlets of *S. acmella* [37].

Phenolics (vanillic acid, *trans*-ferulic acid and *trans*-isoferulic acid) and stigmasteryl glucoside are the active metabolites found in *S. acmella* that elicit a strong antioxidant activity [38]. Literature reveals that the bioactive compounds from all parts of *S. acmella* possess remarkable pharmacological activities. Pharmacologically, *S. acmella* exhibits diverse bioactivities including local anaesthetic and antipyretic [39], diuretic [40], antifungal [41], antiplasmodial [42], antimicrobial [43], and insecticidal activities [44]. Therefore, research studies were carried out to discover plants with antioxidant and anti-inflammatory potentials that could treat various kinds of injuries or protect against diseases. *S. acmella* also exhibited potent anti-inflammatory and antioxidant activities that may contribute to the plant therapeutic value. Table 2 and Figure S1 show phytochemicals and structures of phytochemicals found in *S. acmella.*

## 3. Anti-Inflammatory Effects of *S. acmella*

The inflammatory reaction can be acute (rapid and prompt), chronic (persistent), local (limited to a specific region), or systemic (extended to the whole organism) [51]. The release of cell-derived mediators, such as cytokines, prostaglandins, and reactive oxygen species (ROS) in the acute inflammatory response, helps to protect cells and tissues. ROS such as nitric oxide (NO) and hydroxyl radical, which are physiologically produced from various types of immune cells or respiratory burst in neutrophils, play a role in preventing pathogen invasion, depleting malignant cells, and improving wound healing [52]. However, if inflammatory reaction persists for a longer period than usual, the second stage of inflammation or chronic inflammation may set in, which could predispose individuals to various chronic illnesses [53].

The overproduction of mitochondrial ROS could promote the synthesis of pro-inflammatory cytokines through the activation of the nucleotide-binding domain, leucine-rich repeat-containing family (NLR), and pyrin domain-containing 3 (NLRP3) inflammasomes. NLRP3 induces the elevated release of inflammasomes in response to cytoplasmic ROS [52]. Inflammasomes are a set of intracellular protein complexes that enable autocatalytic activation of inflammatory caspases, leading to host and immune responses by releasing cytokines into circulation [54]. It may trigger the release of IL-1β cytokine from the cytoplasm into the extracellular environment and subsequently activates toll-like receptor (TLR)-1 mediated inflammatory signaling. The activation of TLR-1 by IL-1 triggers NF-κB-activated and mitogen-activated protein kinase (MAPK)-induced pro-inflammatory signaling transductions, producing cytokines, such as IL-1β, IL-6, IL-8, TNF-α, and IFN-γ. A series of these reactions could lead to the amplification of inflammatory events resulting in systemic inflammation.

Medicinal plant species that have been used traditionally to treat pain may have good anti-inflammatory activity [55]. The anti-inflammatory effects of *S. acmella* have been demonstrated by in vitro and in vivo experiments by using several models as summarized in Table 3.

### 3.1. In Vitro Study

Spilanthol, the major pungent compound in *S. acmella* was studied for its anti-inflammatory activities. Wu et al. [56] demonstrated that the anti-inflammatory effects of spilanthol on lipopolysaccharide-activated murine macrophage model, RAW 264.7. The results suggested that spilanthol can inhibit pro-inflammatory mediator production at the transcriptional and translational levels. Spilanthol (90 µM and 180 µM) was found to inhibit NO production, through inhibition of inducible nitric oxide synthase (iNOS) protein expression and iNOS gene transcription alteration. Natural compounds or herbal extracts could exhibit anti-inflammatory activities via the inactivation of NF-κB or activator protein 1 (AP-1) [57]. Spilanthol markedly reduced the inflammatory cytokines such as IL-1β, IL-6, and TNF-α, inhibited the expression of COX-2. This could be partly due to the inactivation of NF-κB. The authors stated that one of the underlying anti-inflammatory mechanisms of *S. acmella* is via the downregulation of NF-κB [56].

The possible mechanism by which *S. acmella* executed the anti-inflammatory action was elucidated in cultured RAW 264.7 cells [58]. *S. acmella* was found to suppress the nuclear localization of NF-κB and expression of NF-κB-dependent cytokines genes, which supported the findings of a previous study by Wu et al. [56]. In inflammatory disease models, activation of Nrf2 has also suppressed inflammation [59]. Recent literature revealed that bioactive compounds including sesquiterpenes [60], flavonoids [61], vanillic acid [62], *trans*-isoferulic acid [63] and scopoletin [64] could activate Nrf2. Since these compounds can be found in *S. acmella* extract, this may indicate its underlying anti-inflammation mechanisms. In addition to the suppression of NF-κB, *S. acmella* was also associated with an increased level of Nrf2 in the nucleus and expression of Nrf2-dependent genes [58].

### 3.2. In Vivo Study

A preliminary study on anti-inflammatory and analgesic activities of *S. acmella* was carried out by Chakraborty et al. [65] in an experimental model of acute inflammation. The authors have used Carrageenan as one of the standard phlogistic agents for testing anti-inflammatory drugs, to induce hind paw edema in a rat. Carrageenan is antigenic but devoid of any apparent systemic effects. The results demonstrated that aqueous extract of *S. acmella* (100 mg/kg, 200 mg/kg, and 400 mg/kg) significantly suppressed carrageenan-induced paw edema and increased the pain threshold in the experimental animal. The anti-inflammatory and analgesic activities of aqueous *S. acmella* extract may be attributed to the presence of flavonoids, which are known to target prostaglandins during the late phase of acute inflammation and pain perception [65]. Inflammatory cells tend to express enzyme COX-2 when activated by the MAPK pathway, which catalyzed the production of prostaglandins (PGs) from arachidonic acid. Overexpression of COX-2 was associated with inflammation and increased PGs production. Flavonoids may interact with the catalytic activity of COX-2 by binding irreversibly to the active site, leaving COX-2 inactive to bind arachidonic acid [66].

Another study by Kim et al. [58], showed the suppression of lung inflammation by methanol extract of *S. acmella* (1 mg/kg and 10 mg/kg body weight) in an acute lung injury mouse model. The results demonstrated that intratracheal *S. acmella* administration effectively relieved acute inflammatory lung disease by suppressing neutrophilic inflammation in lipopolysaccharide-induced lung injury. This was indicated by the reduction of pro-inflammatory cytokines, including IL-1β, IL-6, and TNF-α as well as the suppression of neutrophils infiltration, which is the hallmark of acute lung inflammation.

Huang et al. [70] demonstrated that spilanthol (75 µM to 150 µM) downregulate COX-2 production and decrease TNF-α and MCP-1 production in IL-1β- stimulated lung epithelial cells. The phosphorylation of IκBα and MAPK pathways significantly decreased as compared to IL-1β alone. Spilanthol (>100 µM) promoted HO-1 protein expression by suppressing NF-κB activation and MAPK pathways in IL-1β-activated human lung epithelial cells. Therefore, the author suggested that spilanthol inhibited the expression of the pro-inflammatory cytokines, COX-2, and ICAM-1 by inhibiting the NF-κB and MAPK signaling pathways in human lung epithelial A549 cells. The author also concluded that spilanthol is a natural anti-inflammatory agent. Spilanthol acted as a regulatory factor in MAPK pathways and NF-κB activation of COX-2 and ICAM-1 expression.

A recent study conducted by Bakondi et al. [67] investigated the effects of different parts of *S. acmella* (flower, leaf, and stem) (12.5 µg/mL to 100 µg/mL) in methanol extracts on RAW264.7 inflammatory macrophages. The results showed that all three parts, especially the flowers, significantly suppressed NO production in RAW macrophages exposed to interferon-γ and lipopolysaccharide. The authors indicated that spilanthol (10 µM to 100 µM) of *S. acmella* was responsible for the NO-suppressive effects and provided protection from NO-dependent cell death. The expressions of iNOS mRNA and protein were reduced, whereas the activation of several transcription factors, including NF-κB, was inhibited with spilanthol treatment. In the same study, iNOS inhibitory effect was translated into an anti-inflammatory effect in 12-myristate 13-acetate-induced dermatitis and cerulin-induced pancreatitis animal models. The inhibition of iNOS expression and NO production as well as the suppression of inflammatory transcription factors by spilanthol contributed to the anti-inflammatory actions of *S. acmella,* particularly in the dermatitis model. The histological signs of acute inflammation in the contact dermatitis model were ameliorated by spilanthol treatment.

In another study, Huang et al. [68] examined the effects of spilanthol derived from *S. acmella* on atopic dermatitis inflammation-related symptoms in 2,4-dinitrochlorobenzene (DNCB)-induced skin lesions in mice at 5 mg/kg and 10 mg/kg of spilanthol. The authors discovered that serum immunoglobulin E and immunoglobulin G2a levels were reduced, while COX-2 and iNOS expression were suppressed. Topical spilanthol treatment has also caused a reduction in epidermal thickness and collagen accumulation, inhibition of mast cells, and eosinophils infiltration into the skin lesions. The results obtained by the study indicated that topical spilanthol treatment protected against atopic dermatitis skin lesions through the inhibition of MAPK signaling pathways and reduction of inflammatory cell infiltration. These actions were enough to block allergic inflammation. Spilanthol (3 µM to 100 µM) was also found to reduce inflammatory response by downregulating MAPK signaling pathways in 3T3-L1 pre-adipocytes [69]. Literature shows that obesity-induced chronic low-grade inflammation could lead to the development of various metabolic diseases.

Huang et al. [69] demonstrated that spilanthol (5 mg/kg and 10 mg/kg) might help in preventing pre-adipocyte inflammatory responses to reduce obesity-related metabolic diseases. Spilanthol significantly suppressed inflammatory mediator COX-2, promoted anti-inflammatory protein heme oxygenase-1 (HO-1) expression, and blocked the phosphorylation of c-Jun N-terminal kinase (JNK), as well as P38 protein in LPS-stimulated murine pre-adipocytes. Therefore the authors concluded that spilanthol could be a natural anti-obesity agent mainly due to its role in the activation of MAPK signaling.

As a whole, these findings supported that inflammation may be alleviated by *S. acmella* extract and the bioactive compound responsible for this was spilanthol. Systemic inflammation may be prevented by the inhibition of iNOS, inactivation of NF-κB, suppression of inflammatory cytokines, including IL-1β, IL-6, TNF-α, and the inhibition of COX-2. The anti-inflammatory effect of *S. acmella* was also contributed by its role in the downregulation of MAPK signaling pathways. This evidence proposed that *S. acmella* can be therapeutically developed for a broad spectrum of inflammatory disorders.

## 4. Antioxidant Properties of *S. acmella*

Overexposure to various stimuli such as pollutants, drugs, xenobiotics, ionizing radiation, and heavy metal ions may induce excessive accumulation of ROS or depletion of antioxidant capacity that could alter the redox balance and cause oxidative stress. The cellular redox system, which comprises catalase, superoxide dismutase, glutathione peroxidase, glutathione reductase, and peroxiredoxins, is an integrated cellular defense mechanism that maintains the oxidative equilibrium [71]. In the imbalance between oxidant/antioxidant statuses that result from an excess of ROS, the antioxidant system can be overwhelmed. The highly reactive radicals can irreversibly and permanently damage vital biomolecules, including lipids, proteins, and DNA [51]. This could contribute to many pathological conditions, such as cancer, neurological disorders, cardiovascular disease, pulmonary disease, renal disease, and autoimmune disease [72].

Many medicinal plants with great antioxidant potentials were identified and assayed in a cell-free system (chemical method) and a cell system (cellular method) [73,74]. Many methods were used by researchers to prove the antioxidant properties of plants. The methods were phenolic and flavonoid contents (TPC and TFC), 2,2′-azino-bis(3-ethylbenzothiazoline-6-sulfonic acid (ABTS), ferric reducing antioxidant power (FRAP), oxygen radical absorbance capacity (ORAC), cupric reducing antioxidant capacity (CUPRAC), 2,2-diphenyl-1-picrylhydrazyl (DPPH), cellular antioxidant activity (CAA), β-carotene linoleic acid and superoxide anion radical scavenging (SOD) assays [73,75,76,77]. The presence of antioxidants may reduce oxidative stress in cells, and thus are useful in preventing and treating many diseases.

### 4.1. Antioxidant Activity in a Cell-Free System

The significant antioxidant effect exerted by *S. acmella* is illustrated in Table 4. Wongsawatkul et al. [17] studied the antioxidant activity of *S. acmella* and its potential role as a natural vasodilator in the phenylephrine-induced contraction of rat thoracic aorta. *S. acmella* extracts were reported to possess vasorelaxant and antioxidant activities as shown by a partial release of NO from functional endothelial cells and a strong scavenging activity on 2,2-diphenyl-1-picrylhydrazyl (DPPH) assay. The study also demonstrated that *S. acmella* ethyl acetate and chloroform extract had the highest DPPH scavenging activity (*IC*_50_ = 216 µg/mL) and superoxide radical activity (57.92% SOD, 200 µg/mL) respectively. However, ethyl acetate extract showed the most potent radical scavenging activity with immediate vasorelaxant effects.

In another study, Tanwer et al. [78] evaluated the antioxidant activity of *S. acmella* extracts and showed that all parts of the *S. acmella* plant (0.1 mg/mL), which were the callus, root, stem, and leaf, exhibited antioxidative activities that were comparable to butyl hydroxy anisole (BHA), the standard antioxidant. The stem methanolic extract (39.54%) showed the highest superoxide radical scavenging activity, while the leaves (76.42%) showed the highest DPPH radical scavenging activity. *S. acmella* leaves were reported to have the highest phenolic content (52.3 ± 1.6 mg/g) than other plant parts (roots, 32 ± 0.75 mg/g; stem, 38 ± 1.67 mg/g; callus, 29 ± 1.09 mg/g). The authors concluded that *S. acmella* possessed a strong antioxidant activity and it was comparable to a butyl hydroxytoluene (BHT) reference compound.

Nabi & Shrivastava [79] investigated the presence of polyphenol compounds in *S. acmella* leaves via quantification of total flavonoids and their antioxidant activity. In accordance with earlier studies, the leaves of *S. acmella* extract exhibited the most potent antioxidant activity with the lowest *IC*_50_ values at 134.11 µg/mL for DPPH radical scavenging and 104.51 µg/mL for superoxide radical scavenging assays. The authors documented that the strong antioxidant activity of *S. acmella* was due to the high concentration of flavonoids (72.14 QE mg/g) and phenols (84.52 GAE mg/g) in the leaves extract. These studies demonstrated that *S. acmella* could be an important source of natural antioxidants, which might help in preventing the progress of various oxidative stress-related diseases.

A study on bioassay-guided isolation of *S. acmella* from hexane, ethyl acetate, chloroform, and methanol extracts resulted in a diverse group of bioactive compounds, including phenolics, coumarin, and triterpenoids [38]. Both fractions and bioactive compounds exhibited antioxidant properties in SOD and DPPH assays in the range of 11.29% to 81.50% and 1.90% to 96.05%, respectively. The most potent antioxidant (96.05%) in the DPPH assay was from methanol extract fraction cause the isolation of phenolic compound (*trans*-isoferulic acid). Meanwhile, the highest antioxidant activity of ethyl acetate extract fraction in DPPH (82.46%) and SOD (81.50%) assays induced the isolation of the phenolic compound (vanillic acid). It was also observed that fractions from methanol and chloroform elution exhibited antimicrobial activity. This study reports the isolation of 3-acetylaleuritolic acid, vanillic acid, β-sitostenone, scopoletin, *trans*-ferulic acid, *trans*-isoferulic acid, and a mixture of stigmasteryl and β-sitosteryl glucosides that possessed antioxidant and antimicrobial activities.

A recent study conducted by Swargiary et al. [80] evaluated the phytochemical properties and larvicidal activities of *S. acmella* against Aedes aegypti. The results showed that *S. acmella* methanol extract contained high total phenolic content (approximately 67 µg GAE/mg dry weight) and antioxidant activity in DPPH, TBARS, and superoxide anion scavenging activity with *IC*_50_ values of 730 µg/mL, 57 µg/mL, 965 µg/mL and 175.6 µg/mL, respectively. The authors reported that there was a relation between TPC and antioxidant activity. Higher TPC contributed to higher antioxidant activity. The authors suggested that the higher antioxidant activity was due to phenolics’ ability to chelate metal ions during free radical productions. However, there was no positive correlation between antioxidant activity and larvicidal property against *Aedes aegypti*.

Boontha et al. [81] reported the anticancer effect of *S. acmella* extract on MCF- 7 cells. *S. acmella* extracts contained TPC (62.8 ± 5.2 mg GAE/g), TFC (375.6 ± 20.1 mg rutin equivalent/g) and DPPH with *IC*_50_ (1.2 ± 0.1 mg/mL). Cytotoxicity activity on MCF-7 cells showed *IC*_50_ values of 37.1 ± 1.1 µg/mL in 48 h. *S. acmella* inhibited formation of colony cells with *IC*_50_ values of 44.9 ± 1.3 μM and demonstrated an anti-migration effect at the concentration of 50 µg/mL. The authors suggested that the high antioxidant activity of *S. acmella* extract was due to the phenolics and flavonoids contents in the extract that was responsible for its cytotoxic effect.

In addition to that, the antioxidant potential of different extracts (water, methanol, solvent) of *S. acmella* on different parts (stems, leaves, flowers) was studied by DPPH (2,2-diphenyl-1-picrylhydrazyl) free radical scavenging assay [82]. Methanol extracts of all the plant parts exhibited higher antioxidant capacity with *IC*_50_ values that ranged from 67.34 µg/mL to 127.19 µg/mL when compared to acetone and water extracts. Flower methanol extracts showed the highest antioxidant capacity with an *IC*_50_ value of 67.34 µg/mL. The authors suggested that the antioxidant properties of the *S. acmella* might be due to the presence of highly valuable bioactive compounds such as phenolics, coumarins, and triterpenoids.

### 4.2. Antioxidant Activity in Cell System (Cellular Method)

Literature has revealed that antioxidant compounds have derived from natural products, including flavonoids (epigallocatechin-3-gallate and quercetin) and phenols (phenolic acids and carnosic acid) exhibited neuroprotective activity [85]. Suwanjang et al. [83] recently studied the neuroprotective effect of *S. acmella* extracts (hexane, chloroform, ethyl acetate, and methanol) on pesticide-induced neuronal death. Dopaminergic cell lines (SH-SY5Y) were pretreated with *S. acmella* extracts before being exposed to hydrogen peroxide (H_2_O_2_) or primicarb (pesticide). It was observed that cell viability reduction induced by pirimicarb was attenuated by pretreatment of the cells with *S. acmella* extracts. It was also noted that *S. acmella* hexane extract exerted the strongest protective effect in SH-SY5Y cells exposed to H_2_O_2_. The authors suggested that the neuroprotective effect of *S. acmella* extracts resulted from their antioxidant properties.

Other studies by Gay et al. [84] reported that phenolic compound (5 µM) of vanillic acid and *trans*-ferulic acid in *S.acmella* were able to attenuate cell death on SH-SY5, caused by H_2_O_2_-induced toxicity. The study also demonstrated that the ROS level and apoptotic cells were reduced after 24 h treatment of cells with the phenolic compounds. Phenolic compounds were able to upregulate H_2_O_2_-induced depletion of the expressions of sirtuin-1 (SIRT1), activate FoxO signaling, and mediated oxidative stress resistance [84]. The findings suggested that these phenolics might be promising compounds against neurodegenerative disorders. Collectively, these pieces of evidence showed that the antioxidant effect of *S. acmella* was mainly contributed to by the presence of phenolic compounds.

Several antioxidant mechanisms of flavonoids have been identified. Firstly, the inhibition of enzymes responsible for superoxide anion production and ROS generation. Secondly, the scavenging of ROS and lastly, the chelation of metal ions was responsible for producing ROS [86].

## 5. Conclusions

In vitro and in vivo studies have shown that metabolites of *S. acmella* contributed to their anti-inflammatory and antioxidant activities. *S. acmella* could reduce NO release, inhibit inflammatory cytokines (IL-1β, IL-6, and TNF-α), attenuate COX-2 and iNOS expressions, suppress NF-κB and inhibit MAPK signaling pathways. *S. acmella* and its active metabolites contain high TPC and TFC, which contributed to the high antioxidant activities in a cell-free system through chemical method (DPPH, TBARS, and SOD) assays. Few studies have determined the antioxidant activity of *S. acmella* in a cell system (cellular method). Phenolic compounds of *S.acmella* have demonstrated strong protective effects in SH-SY5Y cells that were exposed to H_2_O_2_ by upregulating SIRT1 and FoxO3a expressions and inducing superoxide dismutase and catalase.

Therefore, *S. acmella*, as well as its metabolites, may have a potential role as a natural antioxidant that is viable for the prevention of various life-threatening diseases. Further clinical studies need to be verified for future prevention and treatment of chronic diseases.

## Figures and Tables

**Table 1:** Taxonomic of *S. acmella.*

Kingdom	Plantae
Subkingdom	Tracheobiont
Phylum	Tracheophyta
Division	Magnoliophyta
Superdivision	Spermatophyte
Class	Magnoliopsida
Sub Class	Asteridae
Order	Asterales
Family	Asteraceae
Subfamily	Mimosoideae
Genus	Spilanthes
Species	Acmella

**Table 2:** Phytochemicals in *S. acmella.*

Groups	Parts	Compounds	References
Alkyl amide	Flower	(2*Z*)-*N*-isobutyl-2-nonene-6,8-diynamide**(1)**, *N*-phenethyl-2, 3-epoxy-6,8-nonadiynamide**(2)**, (2*E*,4*Z*)-*N*-isobutyl-2,4-undecadiene-8,10-diynamide**(3)**, (2*E*)-*N*-isobutyl-2-undecene-8,10-diynamide**(4)**, (2*E*,6*Z*,8*E*)-N-isobutyl-2,6,8-decatrienamide/ Spilanthol**(5)**, (2*E*)-*N*-(2-methylbutyl)-2-undecene-8,10-diynamide**(6)**, (2*E*,7*Z*)-*N*-isobutyl-2,7-tridecadiene-10,12-diynamide**(7)**, (2*E*,7*Z*)-*N*-isobutyl-2,7-decadienamide**(8)**, (2*E*,6*Z*,8*E*)-*N*-(2-methylbutyl)-2,6,8-decatrienamide**(9)**, (2*E*,4*E*,8*Z*,10*Z*)-*N*- isobutyl-dodeca-2,4,8,10-tetraenamide**(10)**, undeca-2*E*,7Z,9E-trienoic acid isobutylamide**(11)**	[16,31,32]
Fatty acid esters	Leaf	α–amyrin acetates**(12)**, β–amyrin acetates**(13)**,	[33,45]
Stigmastane	Aerial	(24ξ)-Stigmast-4-en-3-one**(16)**	[38]
Glucoside	Aerial	Stigmasteryl glucoside**(18)**,	
Phenolics	Aerial	Vanilic acid**(17)**, *trans*-ferulic acid**(19)**, Pentacyclic, *trans*-isoferulic acid**(22)**	
Triterpenoid	Aerial, Leaf	β-sitosterone**(23)**, 3-acetylaleuritolic acid **(21)**, Stigmasterol **(15)**	[38,45]
Fatty acids	Whole plant	Lauric acid**(24)**, Myristic acid**(25)**, Palmitic acid**(26)**, Linoleic acid**(27)**	[46]
Essential oils (Fatty alcohols)	Whole plant	Myricyl alcohol**(14)**, (*E*)-2-hexenol**(28)**, 2-tridecanone**(29)**, hexanol**(30)**, (*Z*)-3-hexanol**(32)**	[33,47]
Aromatic quinone	Aerial	2,2,4-trimethyl-1,2-dihydroquinoline**(31)**	[48]
Glycosides	Whole plant	2-*C*-methyl-D-threono-1,4-lactone-3-O-β-D-glucopyranoside**(33)**, 2-*C*-methyl-D-threono-1,4-lactone-2-O-α-D-fructofuranoside**(34)**	[49]
Pyroglutamate	Whole plant	1,3-butanediol,1-pyroglutamate**(35)**, 1,3-butanediol,3-pyroglutamate **(36)**, 2-*C*-methyl-d-threono-1,4-lactone**(37)**, 2-deoxy-D-ribono-1,4-lactone**(38)**, Dendranthemoside A**(39)**, Dendranthemoside B**(40)**, Ampelopsisionoside**(41)**, Icariside B1**(42)**, Benzyl-α-l-arabinopyranosyl-(1→6)-β-D-glucopyranoside**(43)**, Uridine**(45)**, methyl pyroglutamate**(46)**	[49,50]
Coumarine	Aerial	Chicoriin**(44)**, Scopoletin**(20)**	[38]
Essential oils (Terpene)	Flower head	α-pinene**(47)**, Sabinene**(48)**, β-pinene**(49)**, Myrcene**(50)**, D-3-carene**(51)**, Limonene**(52)**, (*Z*)-β-ocimene**(53)**, (*E*)-β-ocimene**(54)**, γ-terpinene**(55)**, terpinen-4-ol**(56)**, β-elemene**(57)**, β-caryophyllene**(58)**, α-humulene**(59)**, germacrene D**(60)**, (*E*)-β-farnesene**(61)**, (*Z,E*)-α-farnesene**(62)**, β-bisabolene**(63)**, β-sesquiphellandrene **(64)**	[28]

**Table 3:** Summary of anti-inflammatory actions of *S. acmella.*

Part of Plant Used	Experimental Model	Major Findings	References
Whole plant, leaves	Carrageenan induced paw edema	Suppressed hind paw edema; increased pain threshold	[65]
Flowers	RAW264.7 cell lines	Inhibited NO, iNOS, COX-2 protein production; reduced pro-inflammatory mediator production (IL-1β, IL-6, TNF-α); reduced NF-κB binding activity	[56]
Whole plant	RAW264.7 cell lines	Suppressed NF-κB nuclear localization, NF-κB dependent cytokine genes; increased Nrf2 level, Nrf2 dependent cytokine genes; suppressed Nrf2 ubiquitination	[58]
Whole plant	LPS-induced lung injury	Suppressed expression of IL-1β, IL-6, TNF-α; suppressed MPO activity	[58]
Flower, leaf, stem	RAW264.7 cell lines	Suppressed NO production; inhibit iNOS mRNA and protein expression; inhibit NF-κB activation	[67]
Flower, leaf, stem	PMA-induced dermatitis; Cerulean-induced acute pancreatitis	Ameliorated histological signs of acute inflammation in contact dermatitis model; reduced leukocyte migration in an acute pancreatitis mouse model	[67]
Active compound (spilanthol)	DNCB-induced atopic dermatitis	Reduced serum IgE, IgG2a levels; suppressed iNOS, COX-2 expression; inhibited MAPK signaling reduced epidermal thickness, collagen accumulation; inhibited mast cells and eosinophils infiltration	[68]
Active compound (spilanthol)	3T3-L1 cells	Suppressed COX-2, phospho-p38, phosphor-JNK; Promoted protein HO-1 expression; inhibited phosphorylation of MAPK	[69]
Active compound (spilanthol)	A549 cells	Downregulate COX-2 production; decrease TNF-α and MCP-1 production; decreased phosphorylation of IκBα and MAPK pathways; promoted HO-1 protein;	[70]

Abbreviations: NO: nitric oxide; iNOS: inducible nitric oxide synthase; COX: cyclooxygenase; IL: interleukin; TNF: tumor necrosis factor; NF-κB: nuclear factor kappa-light-chain-enhancer of activated B cells; Nrf2: nuclear factor erythroid 2; Ig: immunoglobulin; MAPK: mitogen-activated protein kinase; JNK: c-Jun N-terminal kinase; HO: heme oxygenase.

**Table 4:** Summary of antioxidant actions of *S. acmella.*

Part of Plant Used	Experimental Model	Major Findings	References
Aerial parts	Phenylephrine-induced male Sprague-Dawley rats	Chloroform extract—highest SOD activity and vasorelaxation effect; ethyl acetate extract—most potent radical scavenging activity with immediate vasorelaxation effect.	[17]
Callus, Root, Stem, Leaves	Quantitative estimation of primary metabolites and antioxidant activity	Comparable antioxidative activity to BHA (all plant parts); methanol extract—Highest superoxide radical scavenging activity (stem), highest DPPH scavenging activity (leaves).	[78]
Leaves	Quantitative estimation of phytochemicals and antioxidant activity	Ethanol extract—strong antioxidant activity with lowest *IC*_50_ value for DPPH and superoxide scavenging activity.	[79]
Whole plant	Quantitative estimation of phytochemicals and antioxidant activity	Methanol extract—high TPC, DPPH, TBARS, SOD; High TPC contribute to the high antioxidant activity	[80]
Aerial parts	Quantitative estimation of phytochemicals and antioxidant activity	Ethanol extract- TPC and TFC contribute to high DPPH scavenging activity	[81]
Stem, Leaves, Flowers	Quantitative estimation of phytochemicals and antioxidant activity	Methanol extract- high DPPH scavenging activity	[82]
Aerial parts	Neuronal cell death in SH-SY5Y cell lines	Attenuation of cell viability reduction in pirimicarb-induced in SH-SY5Y cell lines; hexane extract—strongest protective effect in SH-SY5Y cells induced with H_2_O_2_	[83]
Active compound (vanillic acid, *trans*-ferulic acid)	Neuronal cell death in SH-SY5Y cell lines	Attenuate cell death on SH-SY5Y caused by H_2_0_2_-induced toxicity; upregulate H_2_O_2_-induced depletion of the SIRT1, FoxO3a expressions; induced superoxide dismutase 2, catalase, and induced anti-apoptotic Bcl-2 proteins	[84]

Abbreviations: SOD: superoxide anion radical scavenging activity; BHA: butyl hydroxy anisole; DPPH: 2,2-diphenyl-1-picrylhydrazyl; TBARS: thiobarbituric acid reactive substance; H_2_O_2_: hydrogen peroxide; TPC: total phenolic content; SIRT1: sirtuin-1; FoxO3a: forkhead box O 3a; Bcl-2: B-cell lymphoma 2.

## Supplementary Materials

The following are available online at https://www.mdpi.com/article/10.3390/ijerph18073532/s1, Figure S1: Structure of phytochemicals in S. *acmella*.
