# Supplementary material for: Potential Antioxidant and Anti-Inflammatory Effects of Spilanthes acmella and Its Health Beneficial Effects: A Review
Source: Int J Environ Res Public Health. 2021 Mar 29;18(7):3532. doi: 10.3390/ijerph18073532 (PMC8036807; doi:10.3390/ijerph18073532)
Supplement: Supplementary file 1 [file ijerph-18-03532-s001.pdf]

SUPPLEMENTARY MATERIAL (S1)

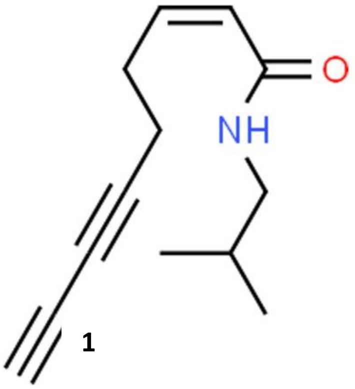

1

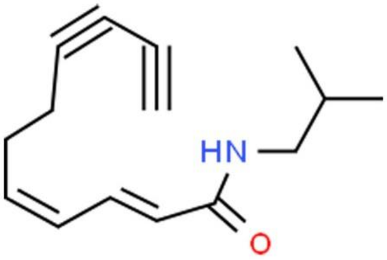

3

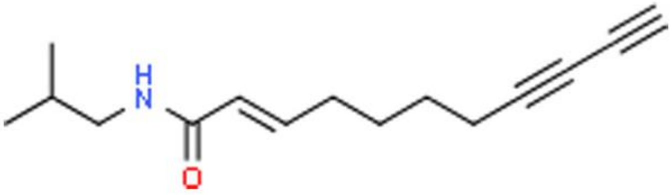

4

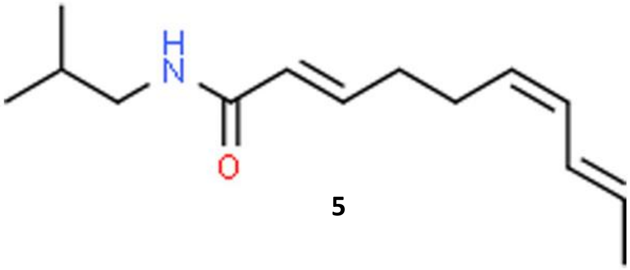

5

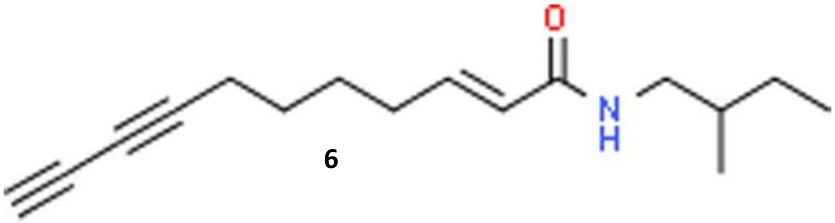

6

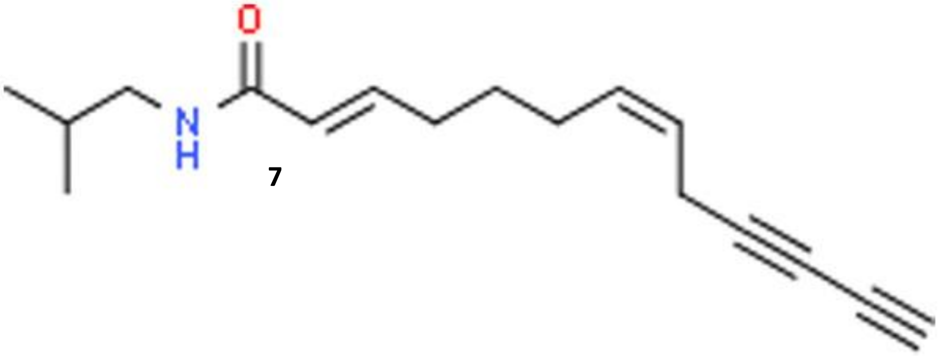

7

Figure S1: Structure of phytochemicals in *S. acmella*

SUPPLEMENTARY MATERIAL (S1)

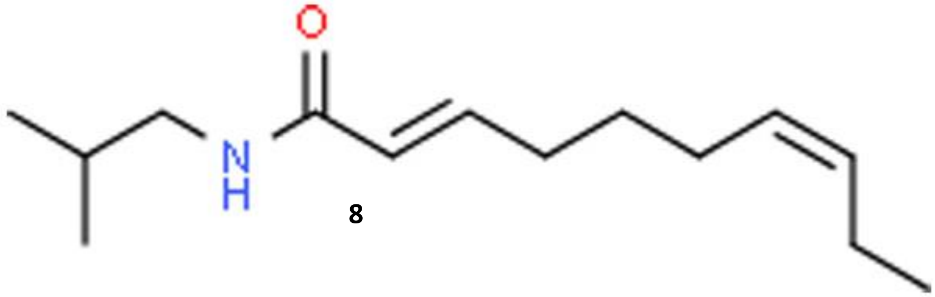

8

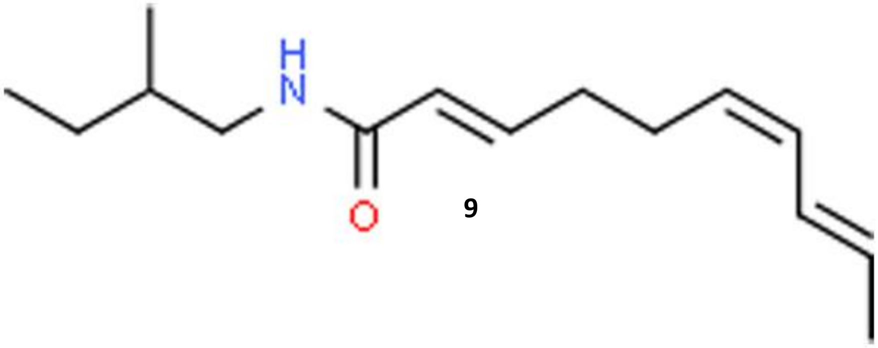

9

10

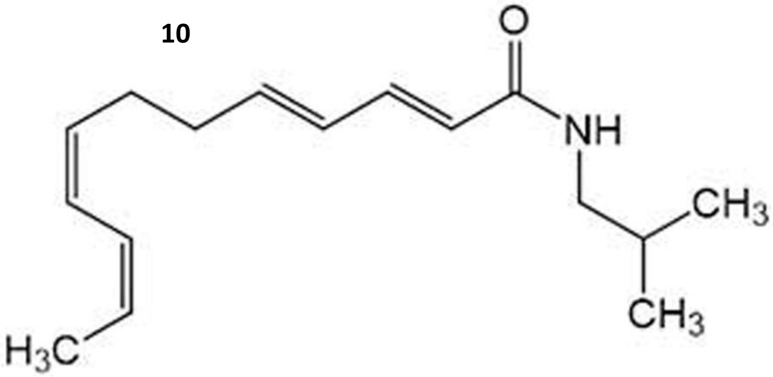

11

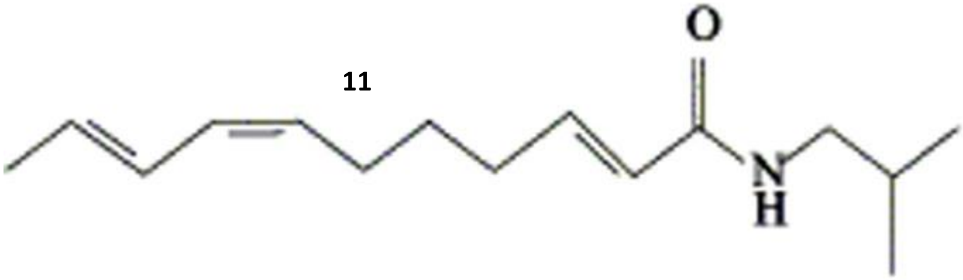

Figure S1: Structure of phytochemicals in *S. acmella*

SUPPLEMENTARY MATERIAL (S1)

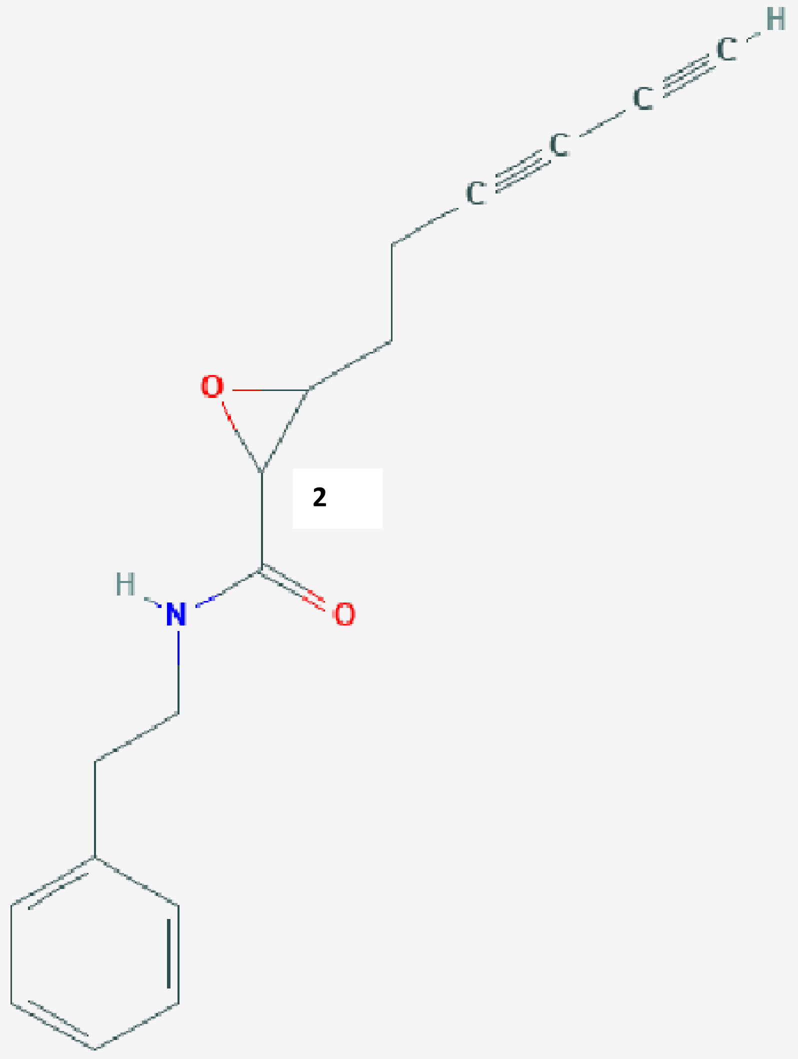

2

20

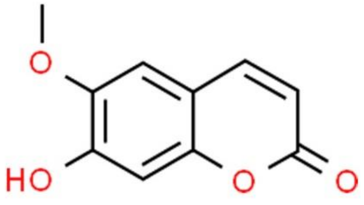

19

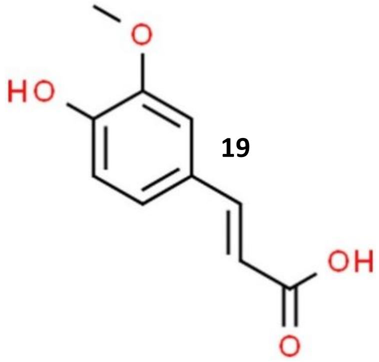

17

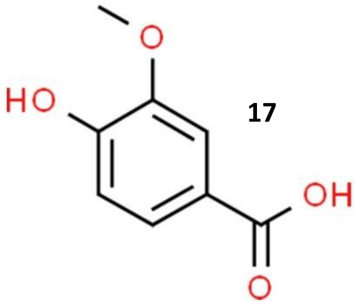

Figure S1: Structure of phytochemicals in *S. acmella*

SUPPLEMENTARY MATERIAL (S1)

14

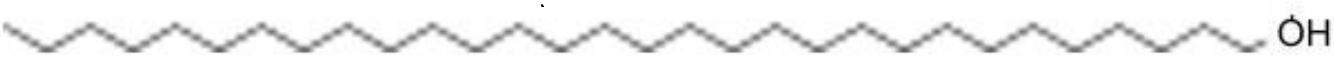

16

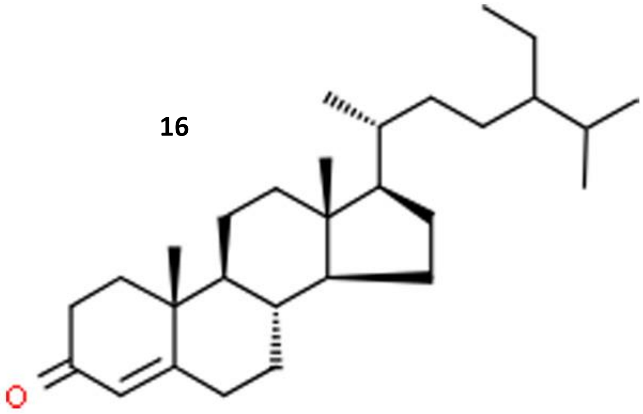

12

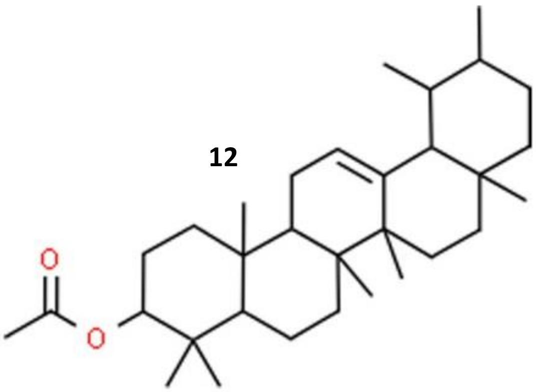

13

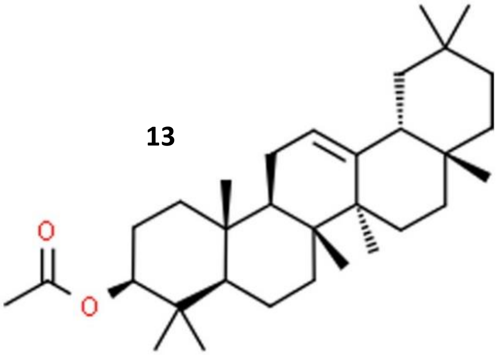

15

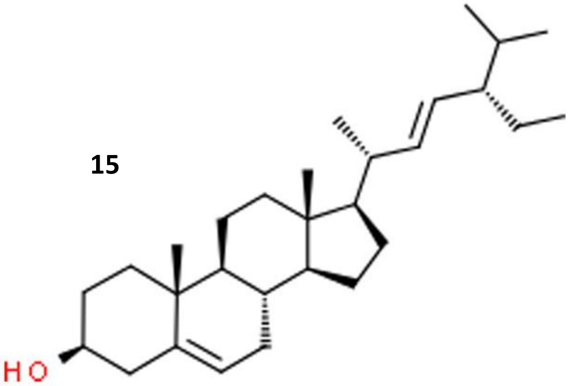

22

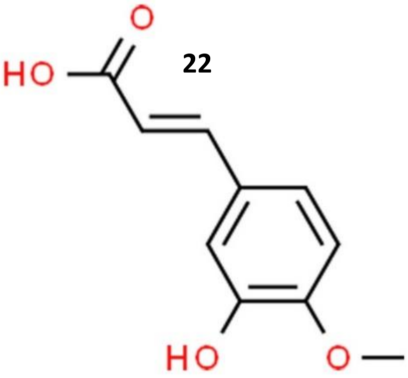

21

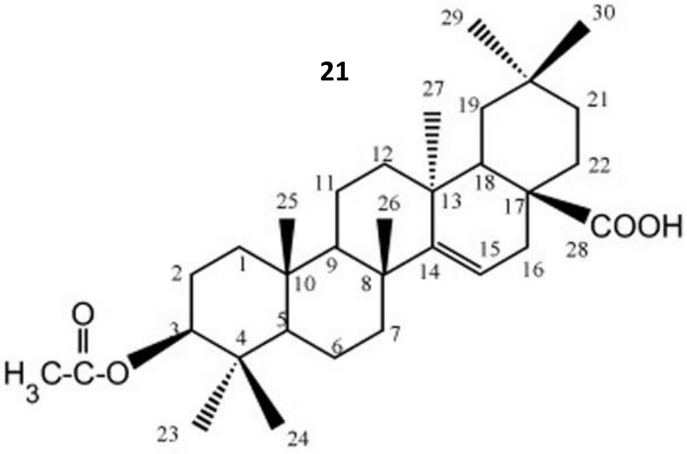

Figure S1: Structure of phytochemicals in *S. acmella*

SUPPLEMENTARY MATERIAL (S1)

27

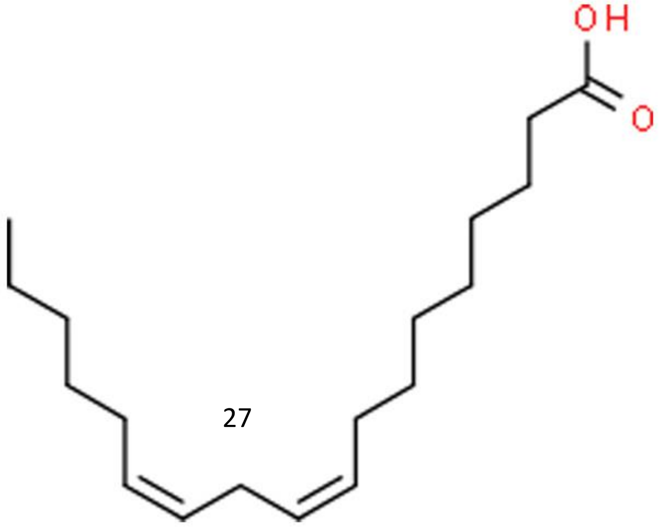

23

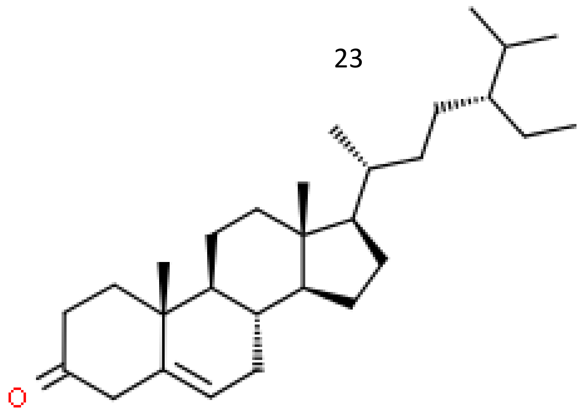

18

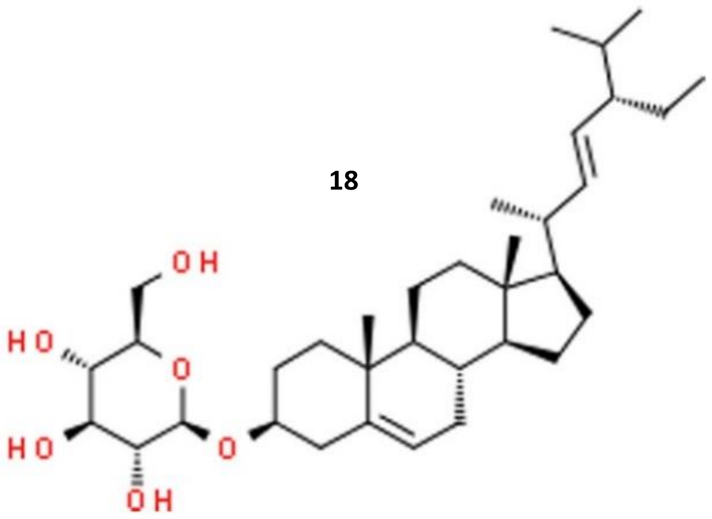

28

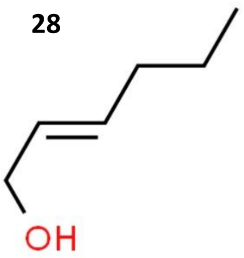

29

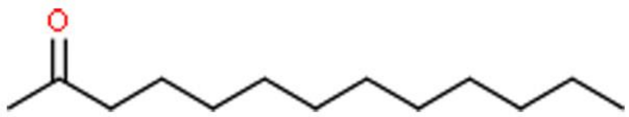

26

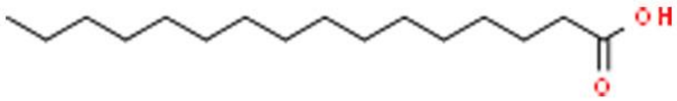

25

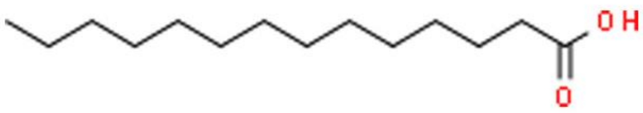

24

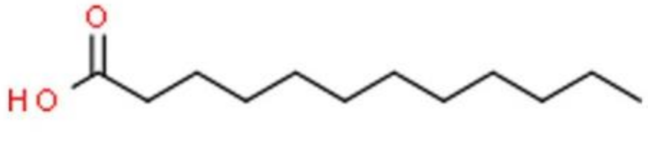

Figure S1: Structure of phytochemicals in *S. acmella*

SUPPLEMENTARY MATERIAL (S1)

30

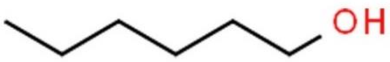

32

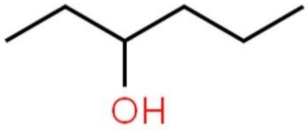

31

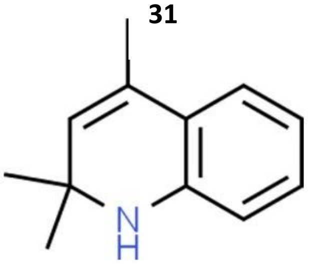

34

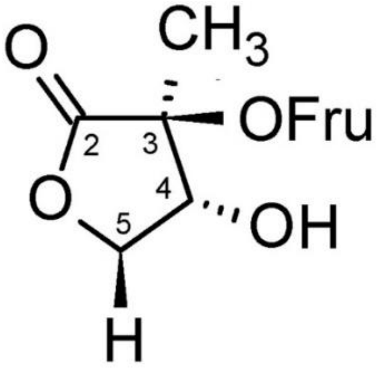

37

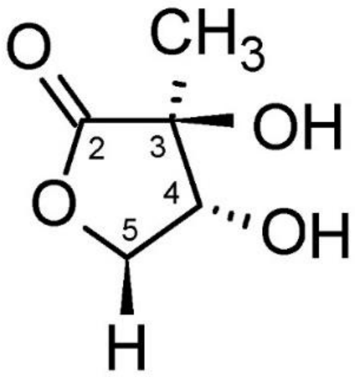

33

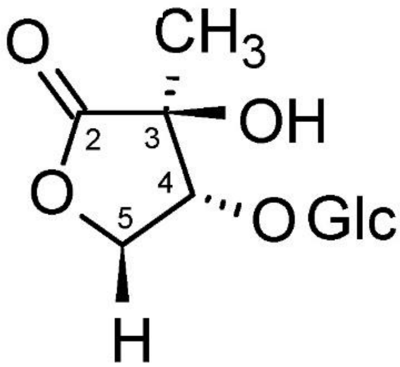

39

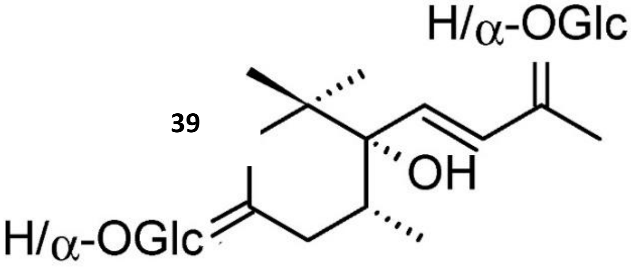

35

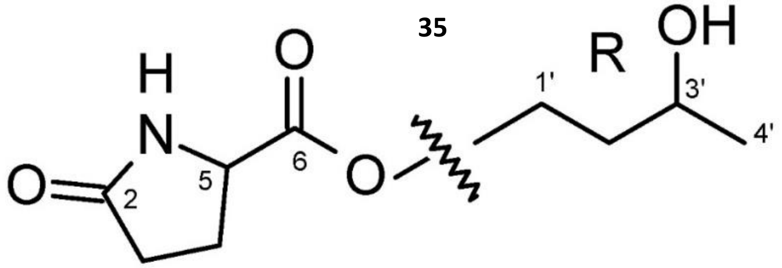

40

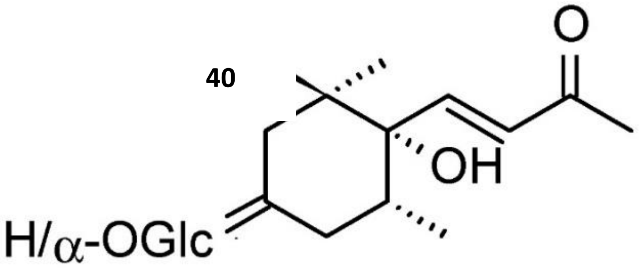

36

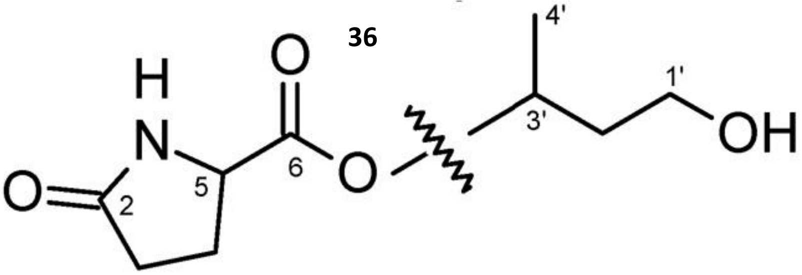

Figure S1: Structure of phytochemicals in *S. acmella*

SUPPLEMENTARY MATERIAL (S1)

38

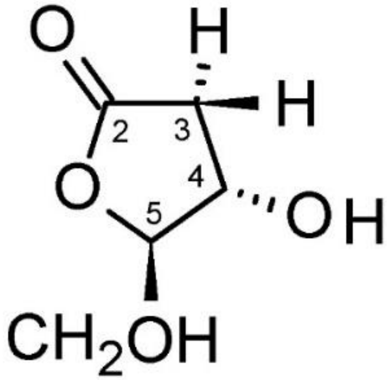

41

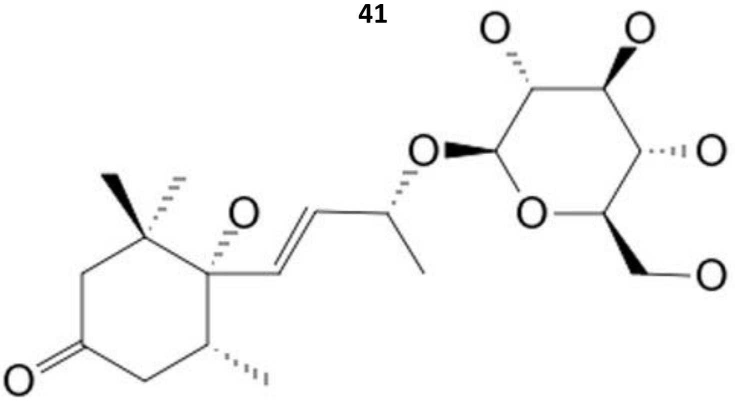

46

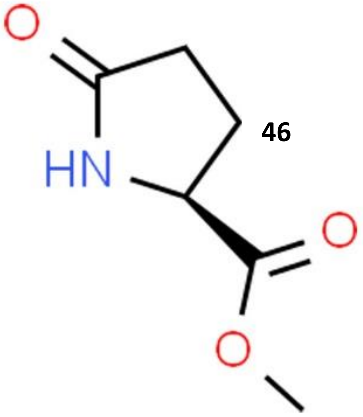

45

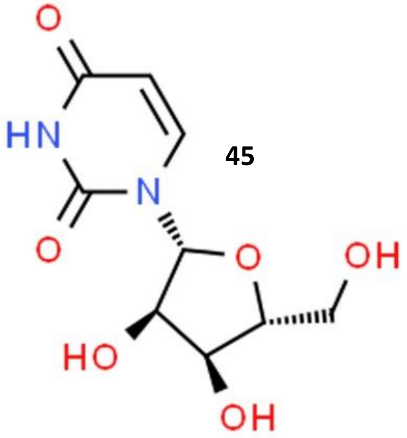

42

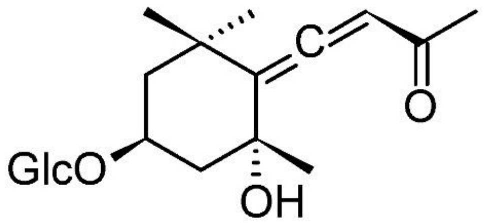

44

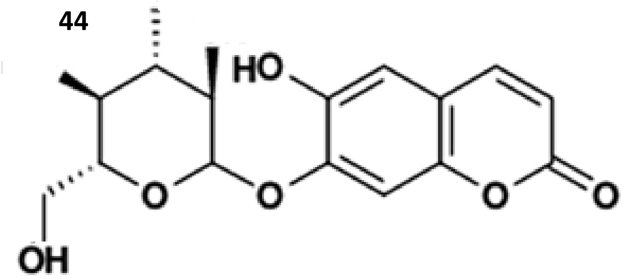

43

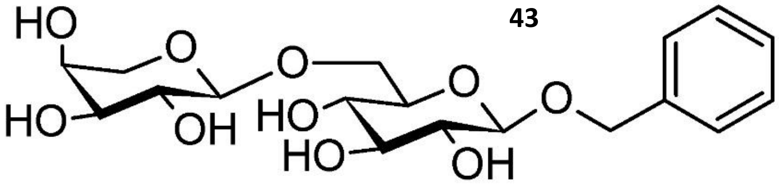

49

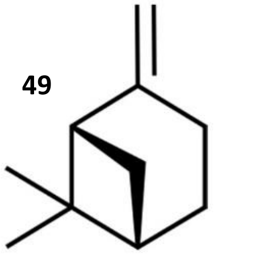

48

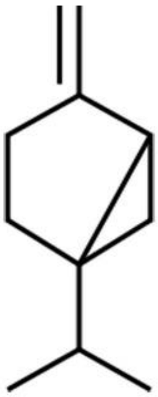

47

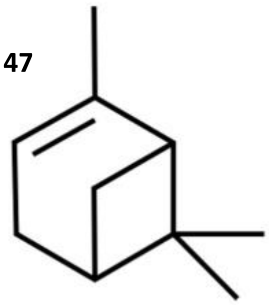

51

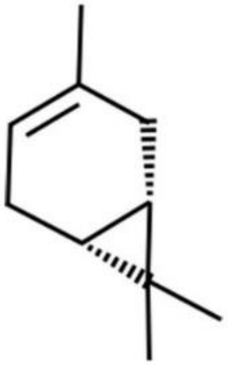

52

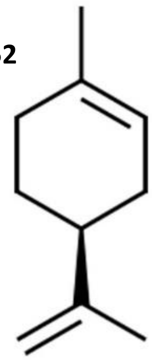

Figure S1: Structure of phytochemicals in *S. acmella*

SUPPLEMENTARY MATERIAL (S1)

50

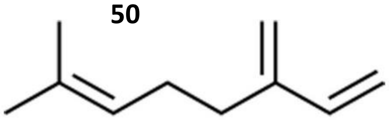

53

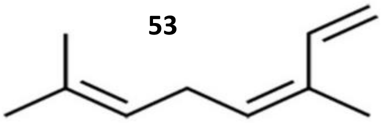

54

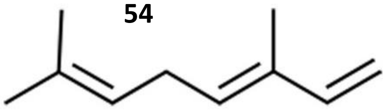

55

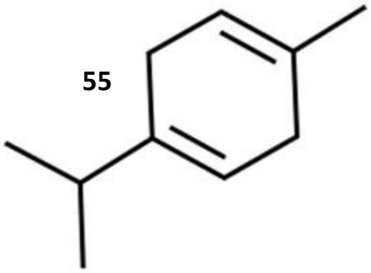

56

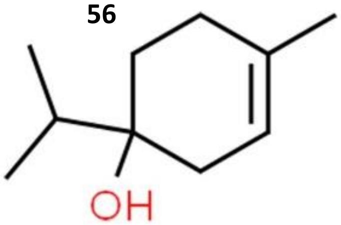

57

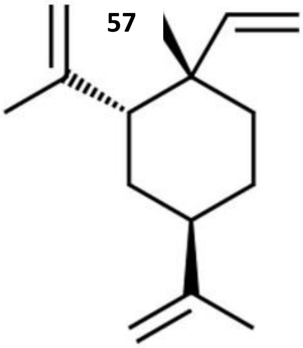

58

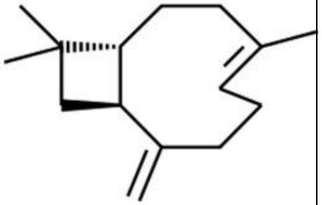

60

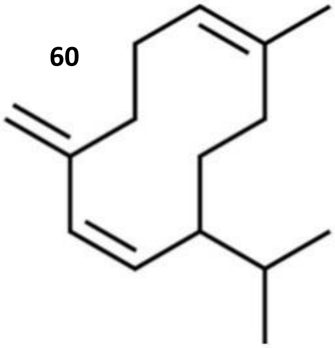

59

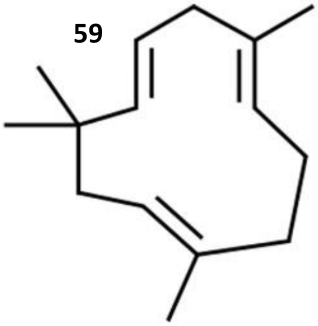

63

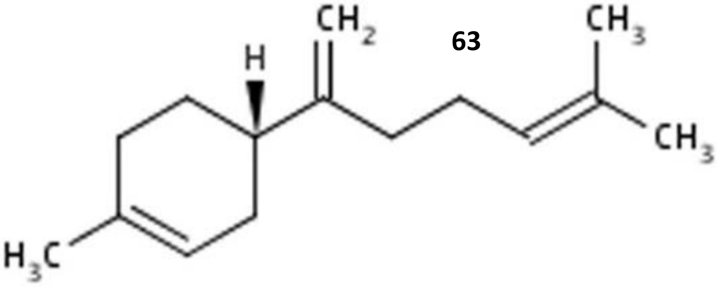

61

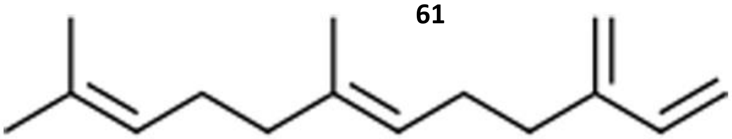

62

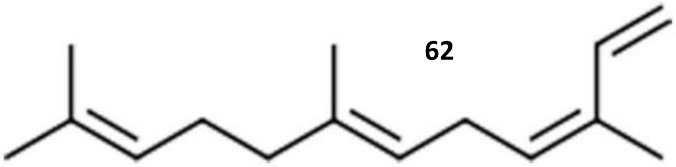

64

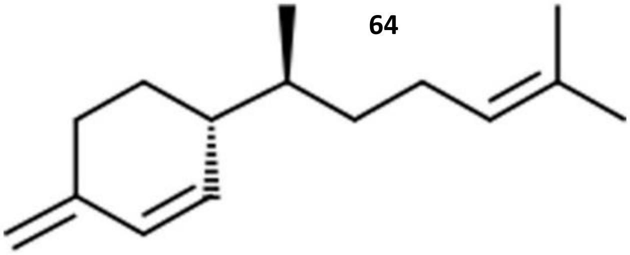

Figure S1: Structure of phytochemicals in *S. acmella*
